# Association of Social Determinants of Health and Health Equity with Adverse Childhood Experiences in Georgia, USA

**DOI:** 10.3390/healthcare14050667

**Published:** 2026-03-06

**Authors:** Gulzar H. Shah, Adverlyn Ivey-Waters, Tobi Oloyede, Shams Rahman

**Affiliations:** 1 Jiann-Ping Hsu College of Public Health, Georgia Southern University, Statesboro, GA 30458, USA; ai03015@georgiasouthern.edu (A.I.-W.); to03842@georgiasouthern.edu (T.O.); 2 College of Global Population Health, University of Health Sciences and Pharmacy in St. Louis, St. Louis, MO 63110, USA; shams.rahman@uhsp.edu

**Keywords:** adverse childhood experiences (ACEs), social determinants of health (SDOH), childhood trauma, physical abuse, sexual abuse, health equity

## Abstract

**Background/Objectives:** Adverse childhood experiences (ACEs), including physical and sexual abuse, are significantly associated with long-term health issues, particularly among socially disadvantaged populations. The study examines the social determinants of health, such as poverty, racial inequities, and limited access to care, to assess their association with adverse childhood experiences, including exposure to physical violence and sexual abuse. **Methods:** We performed multivariable logistic regression analyses using data from the 2023 Georgia Behavioral Risk Factor Surveillance System (BRFSS) (n = 8227) to examine associations between selected ACEs and key social determinants of health (SDOH). **Results:** Our results indicated that a lack of emotional and social support was associated with increased odds of witnessing parental violence (AOR = 2.00) and physical abuse (AOR = 1.90). Absence of food insecurity was associated with lower odds of witnessing parental violence (AOR = 0.65), unwanted sexual touching (AOR = 0.77), and forced sex (AOR = 0.63). Similarly, not reporting transportation barriers was associated with lower odds across ACE outcomes (AORs ranging from 0.54 to 0.65). Sexual and gender minority individuals exhibited substantially higher odds of childhood sexual abuse (AORs = 3.64–5.56). Hispanic ethnicity was associated with increased odds of physical abuse (AOR = 1.47), and older adults (ages 45–64) had greater odds of experiencing forced sex (AORs = 2.08–2.48). These findings highlight complex relationships between SDOH and early trauma. **Conclusions:** Trauma-informed public health strategies must address structural inequities and strengthen emotional and material support for vulnerable populations.

## 1. Introduction

Adverse Childhood Experiences (ACEs) are traumatic events that occur during childhood (before the age of 18), which include physical, emotional, and sexual abuse, as well as neglect and household dysfunction, that can be threatening or harmful [1,2]. These experiences are strongly associated with adverse long-term physical and mental health outcomes, including an increased risk of chronic conditions such as cardiovascular disease, diabetes, depression, and anxiety [3,4,5]. Recent studies have also identified a link between ACEs and cognitive decline in later life, as prolonged childhood exposure to stress can affect brain development and increase vulnerability to neurodegenerative conditions like Alzheimer’s disease [6,7]. The disruption to brain development is primarily in regions such as the prefrontal cortex and hippocampus, which are critical for memory, learning, and executive function [8]. Notably, the cumulative burden of ACEs accelerates cognitive aging, underscoring the importance of early intervention to mitigate these effects [6]. Physical violence and sexual abuse are among the most frequently reported ACEs in Georgia, as children from low-income and race-disadvantaged communities shoulder the immense burden [9,10]. Specifically, these ACEs show that targeted research within the state is needed to fight these disparities. This study is grounded in the Socio-Ecological Model (SEM), which posits that developmental trajectories and health outcomes, including ACEs, are shaped by multiple interconnected factors. Although early research has focused heavily on the individual level of trauma [4,11], modern public health frameworks rely on the socio-ecological model (SEM) to affirm that the environment influences ACEs in context. The Centers for Disease Control and Prevention (CDC) emphasizes that addressing ACEs requires a multilevel approach that examines relationships at the individual, relational (family stability), community (school and neighborhood safety), and societal (social, cultural, and economic policy) levels [12]. As such, the SEM provides a solid foundation for understanding how SHOH functions across these levels to influence childhood trauma.

Equitable access to Social Determinants of Health (SDOH) is critical in addressing disparities in many health outcomes and their proximate determinants, particularly in empowering marginalized populations [13,14,15,16]. According to Healthy People 2030, Social Determinants of Health (SDOH) “are the conditions in the environments where people are born, live, learn, work, play, worship, and age that affect a wide range of health, functioning, and quality-of-life outcomes and risks” [17]. These determinants play a significant role in shaping health equity, defined as the “absence of unfair, avoidable, or remediable differences among groups of people, whether those groups are defined socially, economically, demographically, or geographically or by other dimensions of inequality” [18]. Race, gender identity, and sexual orientation are critical SDOH factors that contribute to disparities in health outcomes, particularly in marginalized populations [19,20]. As such, ignoring these factors perpetuates inequities and obscures the root causes of health disparities. In Georgia, socioeconomic factors such as poverty, housing instability, and limited access to quality education shape exposure to ACEs [10,21]. These conditions are more likely to occur in communities where physical violence and sexual abuse already take place disproportionately in children and disproportionately affect those communities with historical inequities.

There is a growing recognition of the intersection between SDOH and ACEs, particularly regarding race, sexual orientation, and gender identity. Regrettably, marginalized groups are prone to higher exposure to ACEs because of systemic inequities in SDOH [22]. These inequities often place these groups at greater risk for both ACEs and adverse health outcomes later in life. According to Camacho and Henderson, linking SDOH to ACEs requires a critical intersectional approach to fully understand how varying factors shape the impact of ACEs [23]. They further emphasize that children hold vulnerable positions in society, so a lack of proper interventions, programs, and policies can exacerbate high-risk conditions [23]. For instance, in Georgia, communities with systemic racism and high poverty rates often have no access to trauma-informed resources and policies to mitigate childhood trauma. They are more likely to have higher ACEs (physical violence and sexual abuse) [9,22]. People in Georgia face substantial structural barriers that also contribute to health and socioeconomic inequities. High levels of population below poverty, barriers to and disparities in health insurance coverage, widespread medically underserved areas, and a significant shortage in primary care providers disproportionately affect people across the state [24]. The digital divide, exemplified by limited internet and broadband access to some populations and a lack of essential technology, further restricts access to health resources and perpetuates longstanding disparities [24]. Sexual and gender minority individuals in the United States report higher exposure to adverse childhood experiences compared with heterosexual and cisgender peers, a pattern linked to discrimination, stigma, and family rejection. Although recent literature does not specifically document this phenomenon in Georgia, similar structural and social stressors have been described in Southern states, suggesting potential relevance to Georgia [25].

Although research on ACEs exists, a critical gap remains in assessing the role of SDOH in shaping these experiences in Georgia. Existing research has typically explored the relationship between ACEs and their long-term health outcomes, emphasizing how ACEs contribute to mental health conditions and chronic diseases [4,20,23]. Some studies have also examined how SDOH shapes both exposure to ACEs and the effects of ACEs on health nationally [24]. However, research gaps remain in Georgia, where socioeconomic and cultural factors exacerbate these experiences. Despite existing research focusing on the broader Southeastern region [9], more targeted research is needed in Georgia to understand how SDOH fully contributes to ACEs. While existing studies in the Southeastern US have highlighted disparities in the prevalence of ACEs, there is insufficient focus on outlining how particular SDOH, such as economic instability and limited access to mental health resources, impact physical violence and sexual abuse experiences among children in Georgia.

This study addresses some gaps in the existing body of research by focusing specifically on physical violence and sexual abuse in Georgia. Georgia’s high poverty rates, racial inequities, and limited child access to trauma-informed care further make children the most vulnerable population for these traumatic experiences in the unique socioeconomic and cultural context of Georgia [26,27,28]. By focusing on Georgia, this study provides empirical data to support tailored interventions that enhance health equity at the intersection of SDOH and ACEs and to inform policy changes to eliminate childhood trauma disparities. Ultimately, this provides practical implications for public health practitioners and policymakers to mitigate the long-term health burden. Given the profound disparities in health outcomes in the state, this study is essential to understanding how ACEs interact with SDOH. Because ACEs occurred before the social and economic conditions in adulthood, this study does not aim to establish temporal or causal direction. Instead, we examined the associations between SDOH, health equity indicators, and adverse childhood experiences in Georgia. We pursued the following two research questions: (1) Are structural and psychosocial aspects of adulthood associated with the tendency to report childhood exposure to physical violence and sexual abuse? (2) To what extent are sociodemographic factors such as race/ethnicity, sexual orientation, and gender identity associated with Georgia Adults’ tendency to report childhood exposure to physical violence and sexual abuse?

## 2. Materials and Methods

### 2.1. Data Source

This study utilized data from the 2023 Behavioral Risk Factor Surveillance System (BRFSS), an annual cross-sectional survey conducted by the Centers for Disease Control and Prevention (CDC). The BRFSS collects nationally and state-representative data on health-related risk behaviors, chronic health conditions, and the use of preventive services among non-institutionalized U.S. adults aged 18 years and older. Data are collected via random-digit-dialed telephone interviews using a dual-frame approach that includes both landline and cellular telephones, ensuring broad demographic and geographic coverage.

In 2023, BRFSS data were collected from 48 states, the District of Columbia, Guam, Puerto Rico, and the U.S. Virgin Islands. Kentucky and Pennsylvania did not meet the minimum requirements for inclusion in the public dataset. In total, the survey collected responses from more than 400,000 adults using a complex multistage probability sampling design. One adult from each selected household was randomly selected to participate, and survey weighting procedures were applied to enhance representativeness and to account for nonresponse and selection bias. The dataset includes responses to core questions, optional modules, and state-added questions, allowing for both national and state-level analyses.

For this study, analyses were limited to the 8227 respondents from Georgia. The data used in this research were collected prior to the implementation of recent federal policy changes that affect the dataset.

### 2.2. Variables

#### 2.2.1. Dependent Variables

The dependent variables of interest are measuring adverse childhood experiences, i.e., whether or not the study participants experienced any of the following: (a) adults hurt each other, (b) adults hurt me, (c) adults touched me, and (d) adults made me touch them. The survey question, “How often did your parents or adults in your home ever slap, hit, kick, punch, or beat each other up?” measured the variable “adults hurt each other”. The question “Not including spanking (before age 18), how often did a parent or adult in your home ever hit, beat, kick, or physically hurt you in any way?” measured the variable “adults hurt me”. The variable “adults touched me” was operationalized through the survey question “How often did anyone at least 5 years older than you or an adult, ever touch you sexually?” The survey question “How often did anyone at least 5 years older than you or an adult, try to make you touch them sexually?” operationalized the fourth dependent variable “adults made me touch them”. All four dependent variables were dichotomous, coded 0 (no) or 1 (yes). 

#### 2.2.2. Independent and Control Variables

Our inferential analysis consisted of seven independent variables of primary interest: Social and Emotional Support (yes/no), Lost Employment (yes/no), Received food stamps (yes/no), Food that you bought not lasting (yes/no), Not able to pay your mortgage, rent, or utility bills (yes/no), Threatened to shut off services (yes/no), and Lack of reliable transportation (yes/no). The variable for social and emotional support was based on responses to the survey question, “How often do you get the social and emotional support you need?” Responses of “always”, “usually”, “rarely”, and “sometimes” were grouped to indicate having experienced social and emotional support (yes), while everything else was categorized as not experiencing support (no). The response category “rarely” had low frequency and was therefore combined with adjacent categories to ensure adequate cell counts and model stability, while keeping the “no” category separate. The dichotomous variable lost employment (yes/no), was based on the survey question, “In the past 12 months, have you lost employment or had hours reduced?” The dichotomous variable, received food stamps (yes/no), was based on the survey question: “During the past 12 months, have you received food stamps, also called SNAP, the Supplemental Nutrition Assistance Program on an EBT card?” The variable food insecurity in the past 12 months was based on responses to the question, “During the past 12 months, how often did the food that you bought not last, and you didn’t have money to get more?” Responses of “always”, “usually”, “rarely”, “often”, and “sometimes” were grouped to indicate food insecurity (yes). In contrast, other responses were categorized as (no) to indicate no food insecurity in the past 12 months. The dichotomous variable not able to pay your mortgage, rent, or utility bills (yes/no) was based on the survey question, “During the last 12 months, was there a time when you were not able to pay your mortgage, rent, or utility bills?” The dichotomous variable, threatened to shut off services (yes/no), was based on the survey question, “During the last 12 months, was there a time when an electric, gas, oil, or water company threatened to shut off services?” The dichotomous variable, lack of reliable transportation (yes/no), was based on the survey question. “During the past 12 months, has a lack of reliable transportation kept you from medical appointments, meetings, work, or from getting things needed for daily living?”

The control variables are the sociodemographic characteristics such as Sexual Orientations and Gender Identity (Female, straight; Male, straight; All Others), Age Groups (18 to 24 yrs old, 25 to 34 yrs old, 35 to 44 yrs old, 45 to 54 yrs old, 55 to 64 yrs old, 65 or older), Race/Ethnicity (Non-Hispanic Black/African American, Hispanic, Non-Hispanic White, Other), Employment Status (Employed, Not Employed, Retired), Martial Status (Married or Not Currently Married), Annual Income (Less than $15 k, $15 k–$24,999, $25 k–$34,999, $35 k–$49,999, $50 k–$99,999, $100 k–$199,999, $200 k or more), and Received Healthcare Access (Yes or No). The ‘Other’ race category includes non-Hispanic respondents who are not classified as White or Black (e.g., Asian, American Indian/Alaska Native, Native Hawaiian/other Pacific Islander, multiracial, and other race); Hispanic ethnicity is categorized separately. Education level was categorized as Less than or High School Graduate, Attended College, College Graduate, Other; respondents with missing, refused, or indeterminate responses were grouped as “Other” (1.9%) and retained to preserve the sample.

### 2.3. Analysis

For the categorical variables, descriptive statistics—specifically frequencies and weighted percentages—were computed to characterize the survey respondents. A multivariable binomial logistic regression was conducted to examine the associations between independent variables—such as social and emotional support, receipt of food stamps, and other covariates—and four dependent variables representing adverse childhood experiences (ACEs), while controlling for other model factors. Multicollinearity was assessed using the Variance Inflation Factor (VIF), and results indicated low multicollinearity among the independent variables. In the logistic regression models, each ACE outcome was a binary variable, with “yes” indicating the presence of the adverse childhood experience. All analyses were conducted using SAS version 9.4, and survey weights were applied to account for the BRFSS’s complex sampling design.

## 3. Results

### 3.1. Descriptive Results

Table 1 presents the sociodemographic and clinical characteristics of the study population. Among the 8227 Georgia respondents included in this study, 2.5% reported not receiving the social and emotional support they needed, whereas 97.5% reported receiving it. Approximately 9% of respondents reported losing their employment in the past year, and 10.6% reported receiving food stamps. More than one in five participants (22.1%) experienced food insecurity, indicating that the food they purchased did not last, and they lacked the resources to obtain more. Financial instability was also reflected in housing-related indicators: 9.9% could not pay their rent, mortgage, or utility bills, and 6.3% reported receiving shut-off threats from utility providers. A lack of reliable transportation affected 6.7% of participants.

Demographically, the majority of respondents were aged 65 or older (42.0%), with the smallest age group being 18–24 years (5.5%). Most participants identified as Non-Hispanic White (63.5%), followed by Non-Hispanic Black or African American (24.6%), Hispanic (6.1%), and “Other” (5.7%). In terms of sexual orientation and gender identity, over half were female and straight (51.3%), followed by male and straight (38.4%), while 10.3% identified otherwise. Employment status revealed that 44.0% were employed, 20.0% were not employed, and 36.0% were retired. Just over half of the sample were married (50.3%).

Regarding education, nearly two-thirds of respondents (67.3%) were high school graduates or had lower education; 25.6% were college graduates; 5.2% attended some college; and 1.9% were in the Other category (missing, refused, or don’t know). The largest income group was $50,000 to $99,999 (29.6%), followed by $100,000 to $199,999 (20.9%). Only 6.4% earned less than $15,000. Access to healthcare was high: 92.4% reported having access, and 7.6% reported not having access.

### 3.2. Logistic Regression of Adverse Childhood Experiences in Georgia

Multivariable logistic regression analyses were conducted to examine associations between selected adverse childhood experiences (ACEs)—specifically related to household violence and sexual abuse—and demographic and social determinants of health (SDOH). All findings are presented in Table 2.

#### 3.2.1. Your Parents Beat Each Other up

Participants who witnessed parental physical violence had significantly higher odds of this ACE when reporting an absence of social and emotional support (AOR = 2.00, 95% CI: 1.27–3.13). Having no food insecurity (absence of hardship) in the past 12 months was associated with reduced odds (AOR = 0.65, 95% CI: 0.53–0.81). Not reporting reliable transportation as a barrier to healthcare was also significantly associated with lower odds of reporting this ACE (AOR = 0.57, 95% CI: 0.42–0.76). Racial disparities were observed, with individuals identifying as “Other” race/ethnicity having higher odds compared with Non-Hispanic White respondents (AOR = 1.46, 95% CI: 1.07–2.00). Sexual orientation and gender identity (SOGI) disparities were also evident: Odds of reporting this ACE were higher for “Female, Straight” respondents (AOR = 1.40, 95% CI: 1.18–1.65) and “All Others” (AOR = 1.64, 95% CI: 1.24–2.17) compared with Male, Straight respondents.

#### 3.2.2. A Parent Physically Hurt You in Any Way

Among respondents who had been physically harmed by an adult at home, lack of social and emotional support was associated with higher odds (AOR = 1.90, 95% CI: 1.24–2.90). Similarly, not reporting reliable transportation as a barrier to healthcare was also significantly associated with lower odds of reporting this ACE (AOR = 0.54, 95% CI: 0.41–0.71). Compared to respondents having health care access, those reporting no healthcare access had higher odds of reporting physical harm by a parent or adult (AOR = 1.42; 95% CI: 1.08–1.86). Hispanic respondents had higher odds compared with Non-Hispanic White respondents (AOR = 1.47, 95% CI: 1.07–2.01). The SOGI category “All Others” demonstrated elevated odds (AOR = 1.28, 95% CI: 1.00–1.64).

#### 3.2.3. Anyone Ever Touch You Sexually

Not receiving food stamps was associated with lower odds of reporting unwanted sexual touching (AOR = 0.71, 95% CI: 0.53–0.97). Similarly, the absence of food insecurity was associated with lower odds (AOR = 0.77, 95% CI: 0.60–0.99). Not reporting transportation barriers was also associated with reduced odds (AOR = 0.59, 95% CI: 0.42–0.82). SOGI disparities were pronounced: “Female, Straight” respondents had higher odds (AOR = 4.04, 95% CI: 3.22–5.08), and “All Others” exhibited substantially elevated odds (AOR = 5.49, 95% CI: 4.01–7.53). Black, Non-Hispanic respondents had lower odds compared with Non-Hispanic White respondents (AOR = 0.65, 95% CI: 0.52–0.81).

#### 3.2.4. Anyone Ever Force You to Have Sex

Absence of food insecurity was associated with lower odds of reporting forced sexual contact (AOR = 0.63, 95% CI: 0.46–0.88). Not reporting transportation barriers was similarly associated with reduced odds (AOR = 0.65, 95% CI: 0.43–0.98). Age was also a significant factor, with individuals aged 45–54 (AOR = 2.48, 95% CI: 1.28–4.81) and 55–64 (AOR = 2.08, 95% CI: 1.07–4.06) having higher odds than those aged 18–24. SOGI disparities persisted, with elevated odds among “Female, Straight” respondents (AOR = 3.64, 95% CI: 2.58–5.14) and “All Others” (AOR = 5.56, 95% CI: 3.57–8.64).

## 4. Discussion

This study used data from the 2023 Georgia Behavioral Risk Factor Surveillance System (BRFSS) to explore how social determinants of health (SDOH) shape exposure to adverse childhood experiences (ACEs), with a particular focus on physical and sexual abuse. Because ACEs were measured retrospectively and adult SDOH were measured at the time of the survey, the findings should be interpreted as associations rather than evidence of causal relationship. The observed relationships may reflect long-term socioeconomic consequences of childhood adversity, shared structural determinants, or cumulative disadvantage across the life course. This aligns with the literature demonstrating that ACEs are causal antecedents of socioeconomic instability in adulthood [28,29,30].

The findings reinforce a growing body of literature indicating that both structural and psychosocial dimensions of SDOH are critical correlates of childhood adversity. Our findings revealed both expected and unexpected patterns, shedding light on the complex interplay between context, identity, and risk during early life.

Transportation stability (i.e., not reporting transportation as a barrier to accessing healthcare) was consistently associated with lower odds across ACE outcomes. Because transportation barriers were the reference category, adjusted odds ratios below 1.0 indicate that individuals without transportation insecurity had significantly lower odds of reporting ACEs. These findings suggest that transportation instability may reflect broader structural vulnerability rather than functioning as a causal precursor to childhood trauma. These deeper structural deprivations may have included limited access to protective systems such as schools, healthcare facilities, and community-based services [31,32]. Transportation insecurity—recognized as a barrier to health equity—may further isolate marginalized populations from essential resources and social networks [33]. The role of transportation within the ecology of childhood abuse remains underexplored but is consistent with broader frameworks that recognize how environmental and infrastructural deficits compound cumulative disadvantage [34]. Childhood environments marked by instability and restricted mobility may thus reduce opportunities for detection, intervention, or escape from abusive households. This implies that focusing on transportation as an SDOH would have a trickle-down effect, potentially lowering ACE rates by improving access to preventive health measures and support systems.

Consistent with prior research [35], the absence of emotional and social support was significantly associated with increased odds of witnessing parental violence and experiencing physical harm by an adult. Our study shows that limited access to healthcare may indicate broader life hardships, including higher-risk home environments, and the long-term effects of physical harm, such as injuries or chronic health conditions. Emotional neglect is well-established both as a distinct adverse childhood experience (ACE) and as a potentiator of risk for other forms of maltreatment and trauma [36,37]. The present study’s adjusted odds ratios (AORs ≈ 2.0) underscore a psychosocial pathway through which relational deprivation heightens susceptibility to interpersonal violence during childhood. However, the lack of a significant association between emotional support and childhood sexual abuse in our study suggests that intra-household emotional availability alone may be insufficient to explain risk. One plausible explanation is that childhood sexual abuse arises from a multifactorial etiology, involving a complex interplay of individual, relational, and contextual factors [38]. These may include identity-based discrimination [39], grooming tactics [40], or perpetration by individuals outside the primary caregiving environment [40], factors that are less contingent on familial emotional dynamics. This emphasizes how varied strategies are needed: although the interventions of emotional support can reduce the effects of violence-related ACEs, the prevention of sexual abuse needs increased community-level strategies.

Sexual and gender minority (SGM) individuals had significantly higher odds of reporting childhood sexual abuse, consistent with national evidence documenting elevated ACE prevalence in LGBTQ+ populations. These disparities are often attributed to minority stress, discrimination, and familial or community rejection [41,42]. The findings reinforce the need for trauma-informed care that affirms gender and sexual diversity and recognizes identity-based adversity as a social determinant of health. Applying an intersectional lens to these findings, the high AORs (3.64–5.56) suggest that the SGM status is an additional burden to other vulnerabilities, including the racial or economic SDOH, further increasing the risk of trauma. However, the broad confidence intervals for SGM groups (e.g., 3.57–8.64 for forced sex) imply they are less precise, which dampens the power of the associations. Although several subgroup estimates show somewhat wider confidence intervals, these intervals remain reasonably bounded overall, supporting interpretation of the observed associations while still warranting modest caution for the smallest subgroups.

In this study, complex and sometimes counterintuitive patterns emerged in the relationship between adverse childhood experiences (ACEs), race, and some social determinants of health (SDOH). Black respondents were significantly less likely than White respondents to report certain ACEs, diverging from national trends indicating greater cumulative adversity among Black children [43,44]. This discrepancy may reflect differential reporting driven by cultural norms, institutional mistrust, or stigma, rather than true variation in exposure [44,45]. Protective cultural assets such as kinship support and communal resilience may also buffer trauma within Black communities [46]. Notably, food security in the past 12 months was associated with reduced odds of multiple ACEs, including sexual abuse and exposure to parental violence, contrasting with prior research also linking food insecurity to elevated maltreatment risk through mechanisms like caregiver stress and poverty [47,48,49]. Hispanic individuals had significantly higher odds of experiencing physical harm from a parent compared to White individuals. This finding aligns with national data showing elevated ACE exposure in some Hispanic populations, potentially due to structural inequities, underreporting, and limited access to support systems [22]. Culturally tailored interventions are needed to address these disparities. Furthermore, not participating in the Supplemental Nutrition Assistance Program (SNAP) was linked to reduced odds of reporting childhood sexual abuse, although this pattern may reflect underlying socio-economic differences rather than a protective effect of program participation.

### Strengths and Limitations

Strengths of this study included its use of a large, weighted, population-based sample from the 2023 Georgia BRFSS, permitting robust, state-level inference. The analysis employed a multidimensional SDOH framework—which incorporates factors across structural (e.g., transportation, housing instability) and psychosocial (e.g., emotional support) domains—and disaggregated domains. It also disaggregates four distinct ACE outcomes to reduce misclassification and improve etiological specificity. Inclusion of SOGI and race/ethnicity covariates enabled an intersectional analysis of differential ACE risk. Limitations included the cross-sectional design, which precluded causal inference. ACEs were assessed via retrospective self-report, subject to recall and social desirability bias. Several SDOH exposures were measured in adulthood, not during rather than in childhood, potentially creating potential temporal mismatches. Unmeasured confounding from contextual variables (e.g., parental mental illness, neighborhood violence) may bias estimates. Reporting may also have varied across birth cohorts, as societal norms and legal definitions of abuse have evolved. Such shifts in perceptions and cultural norms may have influenced how older respondents interpret and report ACE items. Findings are specific to Georgia and may not generalize to other jurisdictions.

## 5. Addition Information

Combining the “rarely” social support category with adjacent categories may have masked meaningful differences in the level of needed emotional support received, another limitation of this BRFSS-based analysis.

This study underscores the complex connections that come when the survey relies on telephone-based sampling. Consequently, individuals experiencing housing instability or limited phone access may be underrepresented, potentially contributing to selection bias and differential reporting across racial groups. Future studies can address some of these limitations by employing life-course designs using longitudinal data to clarify the temporal ordering among SDOH, poor childhood adversity, and socioeconomic conditions. Those studies may incorporate childhood-context SDOH measures and integrate mixed-methods approaches, including qualitative data, to deepen the understanding of the structural and relational mechanisms linking ACEs and adult social vulnerability.

## 6. Conclusions

Our findings highlight the significance of both structural and psychosocial factors in shaping childhood vulnerability. Transportation instability and inadequate emotional support consistently showed significant association with physical and sexual Adverse Childhood Experiences (ACEs). Our study showed variations by sexual orientation, gender identity, and racial/ethnic groups. Lower odds were observed among respondents not reporting transportation barriers. Observed associations of food security and social support with ACE outcomes suggest that these resources may function as structural and interpersonal markers of stability within the SEM framework. Our findings also underscore the need for trauma-informed, equity-focused public health interventions, in collaboration with leadership and policymakers across SDOH sectors, to mitigate mobility barriers and improve culturally competent services for disproportionately affected communities. Ongoing research employing longitudinal and mixed-methods techniques is essential to elucidate causes, enhance the assessment of childhood problems, and inform interventions customized to Georgia’s distinct socioeconomic and demographic context. Given that multiple industries and governing structures represent social determinants of health, there is a need to strengthen cross-sector collaborations among public health agencies, behavioral health systems, and community-based organizations to improve early detection and prevention of childhood maltreatment.

## Figures and Tables

**Table 1:** Descriptive statistics for sociodemographic and clinical characteristics of study participants.

	Frequency (Unweighted)	Percent (Weighted)
**Received Needed Social and Emotional Support**		
No	156	2.50
Yes	6073	97.5
**Lost Employment**		
No	5589	91.09
Yes	547	8.91
**Received food stamps**		
No	5521	89.41
Yes	654	10.59
**Food insecurity in the past 12 months**		
No	4833	77.86
Yes	1374	22.14
**Not able to pay your mortgage, rent, or utility bills**		
No	5553	90.12
Yes	609	9.88
**Threatened to shut off services**		
No	5781	93.66
Yes	391	6.34
**Lack of Reliable transportation**		
No	5743	93.28
Yes	414	6.72
**Age Group**		
18 to 24 yrs old	456	5.54
25 to 34 yrs old	818	9.94
35 to 44 yrs old	984	11.96
45 to 54 yrs old	1227	14.91
55 to 64 yrs old	1288	15.66
65 or older	3454	41.98
**Race/Ethnicity**		
Non-Hispanic Black/African American	1982	24.62
Hispanic	492	6.11
Non-Hispanic White	5115	63.54
Other	461	5.73
**Sexual Orientation and Gender Identity**		
Female, straight	3922	51.27
Male, straight	2936	38.38
All Others	791	10.34
**Employment Status**		
Employed	3588	44.02
Not Employed	1627	19.96
Retired	2935	36.01
**Marital Status**		
Married	4104	50.33
Not Currently Married	4050	49.67
**Education Level**		
Attended College	430	5.23
College Graduate	2106	25.6
Other (missing, refused, don’t know)	158	1.92
High School Graduate or Lower	5533	67.25
**Annual Income**		
Less than $15 k	411	6.43
$15 k–$24,999	656	10.26
$25 k–$34,999	813	12.72
$35 k–$49,999	833	13.03
$50 k–$99,999	1894	29.63
$100 k–$199,999	1338	20.93
$200 k or more	447	6.99
**Healthcare Access**		
No	591	7.65
Yes	7139	92.35

**Table 2:** Logistic Regression of Adverse Childhood Experiences in Georgia.

	Your Parents Beat Each Other Up		A Parent Physically Hurt You in Any Way		Anyone Ever Touched You Sexually		Anyone Ever Forced You to Have Sex	
	AOR	CI	AOR	CI	AOR	CI	AOR	CI
Receive social and emotional Support (Ref. Category = yes)	**2.00**	1.27–3.13	**1.90**	1.24–2.90	1.16	0.62–2.15	1.29	0.60–2.77
Lost Employment (Ref. Category = yes)	0.96	0.73–1.25	0.87	0.69–1.10	0.79	0.59–1.05	0.72	0.50–1.03
Received food stamps (Ref. Category = yes)	1.12	0.85–1.48	0.86	0.67–1.11	**0.71**	0.53–0.97	0.94	0.64–1.40
Food insecurity in past 12 months (Ref. Category = yes)	**0.65**	0.53–0.81	0.84	0.70–1.02	**0.77**	0.60–0.99	**0.63**	0.46–0.88
Utility Bills: Not able to pay your mortgage, rent, or utility bills (Ref. Category = yes)	1.17	0.87–1.59	1.02	0.78–1.35	0.87	0.62–1.21	0.72	0.48–1.08
Threatened to shut off services	0.73	0.53–1.01	0.91	0.67–1.24	**0.62**	0.44–0.87	0.77	0.51–1.18
Lack of reliable transportation (Ref. Category = yes)	**0.57**	0.42–0.76	**0.54**	0.41–0.71	**0.59**	0.42–0.82	**0.65**	0.43–0.98
Age Group (Ref. Category = 18–24 yrs)								
25 to 34 yrs old	0.96	0.62–1.48	1.02	0.69–1.50	1.19	0.73–1.92	1.42	0.71–2.82
35 to 44 yrs old	1.13	0.74–1.74	0.94	0.64–1.39	1.02	0.63–1.67	1.67	0.84–3.32
45 to 54 yrs old	1.26	0.83–1.92	1.13	0.78–1.65	1.41	0.88–2.25	**2.48**	1.28–4.81
55 to 64 yrs old	1.10	0.72–1.68	1.12	0.77–1.63	1.33	0.83–2.13	**2.08**	1.07–4.06
65 or older	0.99	0.64–1.54	0.98	0.66–1.44	0.84	0.51–1.39	1.10	0.53–2.25
Race (Ref. Category = Non-Hispanic White)								
Non-Hispanic Black/African American	1.10	0.92–1.32	1.08	0.92–1.28	**0.65**	0.52–0.81	0.95	0.71–1.27
Hispanic	1.31	0.92–1.85	**1.47**	1.07–2.01	1.04	0.70–1.54	1.06	0.61–1.83
Other	**1.46**	1.07–2.00	**1.52**	1.15–2.01	0.95	0.65–1.39	0.95	0.55–1.65
Sexual Orientation and Gender Identity (Ref. Category = Male, Straight)								
Female, Straight	**1.40**	1.18–1.65	0.90	0.78–1.04	**4.04**	3.22–5.08	3.64	2.58–5.14
All Others	**1.64**	1.24–2.17	1.28	1.00–1.64	5.49	4.01–7.53	5.56	3.57–8.64
Education Level (Ref. College Graduate)								
Attended college	1.00	0.69–1.45	0.81	0.57–1.15	0.76	0.47–1.21	0.62	0.34–1.14
High School Grad or Lower	0.75	0.38–1.48	0.51	0.26–1.00	0.40	0.16–1.01	0.33	0.09–1.17
Others (missing, refused, DK)	0.77	0.64–0.92	0.96	0.81–1.13	0.96	0.77–1.19	0.76	0.57–1.02
Healthcare Access (Ref. Category = yes)	1.19	0.88–1.61	**1.42**	1.08–1.86	1.16	0.82–1.63	1.18	0.77–1.81

AOR: Adjusted Odds Ratio; CI: Confidence Interval; DK = Don’t Know; AORs in bold font indicate significance at *p* ≤ 0.05.

## Data Availability

Publicly available datasets were analyzed in this study. This data can be found here (Behavioral Risk Factor Surveillance System (BRFSS)): https://www.cdc.gov/brfss/index.html (accessed on 15 January 2025).

## Abbreviations

The following abbreviations are used in this manuscript:

ACEs	Adverse childhood experiences
AOR	Adjusted odds ratio
BRFSS	Behavioral Risk Factor Surveillance System
CDC	Centers for Disease Control and Prevention
CI	Confidence interval
EBT	Electronic Benefit Transfer
IRB	Institutional Review Board
NH	Non-Hispanic
SAS	Statistical Analysis System
SDOH	Social determinants of health
SGM	Sexual and gender minority
SNAP	Supplemental Nutrition Assistance Program
SOGI	Sexual orientation and gender identity
U.S.	United States
VIF	Variance inflation factor
WHO	World Health Organization
